# A Simulation-Based Mechanical System-Identification Framework for Non-Invasive Lung Diagnostics and Personalized Pulmonary Rehabilitation

**DOI:** 10.3390/life16040555

**Published:** 2026-03-27

**Authors:** Paraschiva Postolache, Călin Gheorghe Buzea, Alin Horatiu Nedelcu, Constantin Ghimus, Valeriu Aurelian Chirica, Razvan Tudor Tepordei, Simona Alice Partene Vicoleanu, Ana Maria Dumitrescu, Manuela Ursaru, Emil Anton, Cătălin Aurelian Ștefănescu, Constantin Stan, Sorin Bivolaru, Alexandru Nechifor

**Affiliations:** 1 Faculty of Medicine, Grigore T. Popa University of Medicine and Pharmacy Iasi, 16 Universitatii Street, 700115 Iasi, Romania; par.postolache@umfiasi.ro (P.P.); cosghimus@gmail.com (C.G.); valeriu-aurelian.chirica@umfiasi.ro (V.A.C.); razvan.tepordei@umfiasi.ro (R.T.T.); partene.vicoleanu@umfiasi.ro (S.A.P.V.); ana-maria_dumitrescu@umfiasi.ro (A.M.D.); manuela.ursaru@umfiasi.ro (M.U.); emil.anton@yahoo.com (E.A.); 2 National Institute of Research and Development for Technical Physics, IFT Iași, 700050 Iasi, Romania; calinb2003@yahoo.com; 3 “Prof. Dr. Nicolae Oblu” Clinical Emergency Hospital Iași, 700309 Iasi, Romania; 4 Department of Sports and Physical Education, Faculty of Physical Education and Sport, “Dunarea de Jos” University of Galati, 800008 Galati, Romania; catalin.stefanescu@ugal.ro; 5 Faculty of Medicine and Pharmacy, Research Centre in the Medical-Pharmaceutical Field, “Dunarea de Jos” University of Galati, 800008 Galati, Romania; constantin.stan@ugal.ro; 6 Medical Clinical Department, Faculty of Medicine and Pharmacy, “Dunarea de Jos” University of Galati, 800008 Galati, Romania; sorin.bivolaru@ugal.ro; 7 Department of Individual Sports and Physiotherapy, Faculty of Physical Education and Sport, “Dunarea de Jos” University of Galati, 800008 Galati, Romania; alexandru.nechifor@ugal.ro

**Keywords:** lung mechanics, mechanical system identification, inverse analysis, frequency response, viscoelastic modeling, regional lung stiffness, non-invasive diagnostics, pulmonary disease modeling, mechanical impedance, robustness analysis

## Abstract

Current diagnostic assessments of lung disease rely primarily on medical imaging and global pulmonary function tests, which either provide static structural information or collapse complex regional behavior into global indices. As a result, important information about regional mechanical heterogeneity and early pathological changes may remain inaccessible. In this work, we introduce a conceptual diagnostic framework for the lung based on mechanical system identification and evaluate its feasibility using simulation-based analysis. Rather than directly imaging internal lung structure, the lung–thorax system is treated as an identifiable viscoelastic dynamical system whose internal mechanical properties can be inferred from its response to controlled external excitation. A multi-degree-of-freedom mechanical representation of the lung was developed to capture the dominant low-frequency behavior of the chest wall and major lung regions. Sensitivity and Fisher-information analysis confirmed the structural identifiability of regional stiffness parameters (FIM eigenvalues λ_1_ = 1.75 × 10^−9^ and λ_2_ = 8.91 × 10^−10^). Inverse fitting experiments accurately recovered simulated stiffness perturbations (e.g., *k*_01_ = 240 → 239.5; *k*_02_ = 154 → 159.5) from noisy frequency response data, while classification experiments achieved the complete separation of simulated pathological configurations in an idealized synthetic scenario, supporting theoretical discriminability rather than clinical performance claims. These findings demonstrate the theoretical feasibility of a diagnostic paradigm in which regional lung mechanical alterations can in principle be identified through mechanical system identification rather than direct imaging, thereby suggesting a complementary approach for a non-invasive assessment of regional lung mechanics from externally measured responses. By quantifying regional stiffness and mechanical heterogeneity, this framework may also support the personalization and monitoring of pulmonary rehabilitation strategies in chronic respiratory disease.

## 1. Introduction

Assessment of lung function is central to diagnosing and managing respiratory diseases. Traditional pulmonary function tests such as spirometry and body plethysmography provide global measures of airflow, volumes, and volumes changes [1,2,3,4], but they cannot directly resolve the regional mechanical heterogeneity of the respiratory system. To probe mechanical properties beyond global indices, clinicians and researchers increasingly use oscillatory techniques that apply external perturbations to the respiratory system and record the resulting pressure–flow responses to estimate mechanical impedance [5,6,7,8,9].

The forced oscillation technique (FOT) and its variants—including impulse oscillometry—were introduced to measure respiratory system impedance non-invasively by superimposing small-amplitude oscillations during spontaneous breathing [5,7,10,11]. In these methods, the applied oscillatory pressure or flow at multiple frequencies allows the computation of respiratory resistance and reactance as functions of frequency, which reflect elastic, resistive, and inertial components of lung and chest wall mechanics [5,6,8,12]. This frequency-dependent analysis offers advantages over traditional tests because it requires minimal subject effort and can detect abnormalities in lung mechanics that correlate with disease severity [9,11,13,14]. However, despite their clinical utility, current oscillatory and impedance measures typically yield summary indices that are limited in spatial resolution and often insensitive to underlying regional heterogeneity [7,15,16,17,18].

Respiratory system mechanics has also been modeled as lumped networks of mechanical elements to interpret impedance measurements in healthy subjects and in disease states such as asthma, chronic obstructive pulmonary disease (COPD), and interstitial lung diseases [12,19,20,21,22,23]. These models often analogize the lung and chest wall to combinations of resistors, springs, and inertial elements (akin to electrical RLC circuits), enabling an estimation of effective mechanical parameters from measured data [20,23,24]. Yet, existing models are typically applied on a case-by-case basis and usually do not leverage the full dynamical information contained in the frequency response across a broad range of actuations.

In many engineering fields, system-identification methods treat a physical system as a dynamical object, actively excite it with controlled, known inputs, and infer internal parameters from the measured response. Such approaches are foundational in structural health monitoring and vibration-based diagnostics because they exploit the entire dynamic response rather than isolated summary measures [16,18,25,26,27]. In contrast, lung diagnostics has rarely adopted this system-identification paradigm explicitly, even though respiratory mechanics is fundamentally a dynamical system governed by differential equations relating tissue elasticity, mass, and damping [5,8,16,23,25].

In parallel, imaging modalities such as chest radiography and computed tomography provide detailed structural information but are inherently static and do not directly quantify regional mechanical behavior [15,17,18,28]. Lung ultrasound is increasingly used as a bedside tool, but its diagnostic value relies largely on artefact interpretation at air–tissue interfaces rather than on direct measurement of tissue mechanics [29]. Consequently, neither imaging nor standard functional tests offer a direct, model-based route to regional mechanical characterization of the lung–thorax system [15,17,18,28].

Pulmonary rehabilitation is a cornerstone of evidence-based care for chronic respiratory disease and is strongly recommended for patients with chronic obstructive pulmonary disease (COPD, bronchial asthma, etc., including those with an occupational component) and interstitial lung disease by contemporary clinical practice guidelines [30,31,32,33,34]. In these programs, structured exercise training, education, and behavior change aim to improve exercise tolerance, symptoms, and health-related quality of life. However, patients entering the same rehabilitation program often exhibit markedly different underlying mechanical phenotypes, including regional fibrosis, emphysema, or chest wall abnormalities that are not captured by global spirometric indices alone [30,31,32,33]. The absence of a non-invasive, model-based method to quantify regional lung mechanics currently limits the ability to tailor rehabilitation intensity, modality, or monitoring to individual patients. A diagnostic framework that provides physically interpretable parameters, such as regional stiffness and coupling, could therefore inform personalized pulmonary rehabilitation by identifying mechanical targets, anticipating heterogeneous response, and supporting longitudinal tracking of mechanical adaptation.

Although the present work builds on decades of research on respiratory oscillometry [5,6,7,8,9], the diagnostic framework proposed here is fundamentally distinct from conventional forced oscillation techniques. In standard FOT and impulse oscillometry [5,7,10,11,13,14], oscillatory pressure or flow is applied at the airway opening, and the response is summarized as respiratory system impedance, typically interpreted through global resistance and reactance indices or low-order compartment models. In contrast, the approach introduced here treats the lung–thorax complex explicitly as a coupled multi-degree-of-freedom mechanical system that is externally excited at the chest wall and identified through its full frequency-domain response. Rather than estimating impedance at a single boundary, the objective is to infer internal regional mechanical parameters by exploiting mode coupling and frequency-dependent dynamics [15,16,19,23,25]. This distinction shifts the diagnostic focus from impedance characterization to mechanical system identification, enabling regional inference from externally measured surface responses.

Here, we propose a conceptual diagnostic framework for the lung based on mechanical system identification and evaluate its feasibility using simulation. Instead of using static imaging or global indices, we treat the lung–thorax complex as a coupled viscoelastic dynamical system whose internal properties can be inferred from externally measured responses to controlled external excitation. Mechanical changes associated with pathology—such as regional stiffness alterations or heterogeneity—are reflected in frequency-domain response functions and, in principle, can be identified through inverse analysis. To demonstrate feasibility, we construct a multi-degree-of-freedom mechanical representation of the lung and show through simulation that pathological changes produce distinguishable signatures in the system response. This model establishes a new diagnostic conceptual framework whereby disease is inferred from dynamical behavior rather than from static snapshots or single scalar indices. In this sense, the proposed method shifts lung diagnostics from impedance measurement to inverse mechanical characterization.

## 2. Materials and Methods

### 2.1. Mechanical Lung Model and System-Identification Framework

#### 2.1.1. Modeling Approach and Assumptions

The lung–thorax system was modeled as a linearized, low-frequency viscoelastic dynamical system, suitable for small-amplitude external excitation. The model is intended to capture the dominant mechanical behavior of the chest wall and major lung regions under gentle perturbations, such as low-intensity acoustic or vibrational excitation, while remaining analytically tractable for inverse analysis.

The following assumptions were adopted:

1. Small-signal regime: displacements are sufficiently small to justify linearization.
2. Low-frequency excitation: analysis is restricted to frequencies below approximately 200 Hz, where inertial, elastic, and viscous effects dominate and wave propagation effects can be neglected.
3. Lumped-parameter representation: distributed lung and chest wall properties are represented by effective lumped parameters.
4. Passive mechanics: active muscle contraction and nonlinear airflow effects are neglected.
5. Isotropic effective properties: each lung region is characterized by averaged mechanical parameters.

These assumptions are standard in mechanical impedance modeling, elastography, and low-frequency structural dynamics and are appropriate for a proof-of-concept investigation [5,6,8,12,23,24,29].

The present assumptions should therefore be interpreted as valid primarily for passive, small-amplitude, low-frequency probing of the lung–thorax system, where global and regional viscoelastic behavior dominates over nonlinear airway phenomena, recruitment/derecruitment effects, and propagating wave dynamics. The model is not intended to represent forced breathing, large deformation, active respiratory effort, or strongly frequency-dependent tissue behavior.

#### 2.1.2. Degrees of Freedom and Physical Compartments

A four-degree-of-freedom (4-DOF) model was employed to represent major mechanical compartments of the thorax (see Table 1).

Each degree of freedom is associated with a generalized displacement coordinate

$$x_{i}\left(t\right),\;\;\;\;i=0,\;1,\;2,\;3,$$

representing effective modal motion rather than literal anatomical displacement.

**Figure 1:**
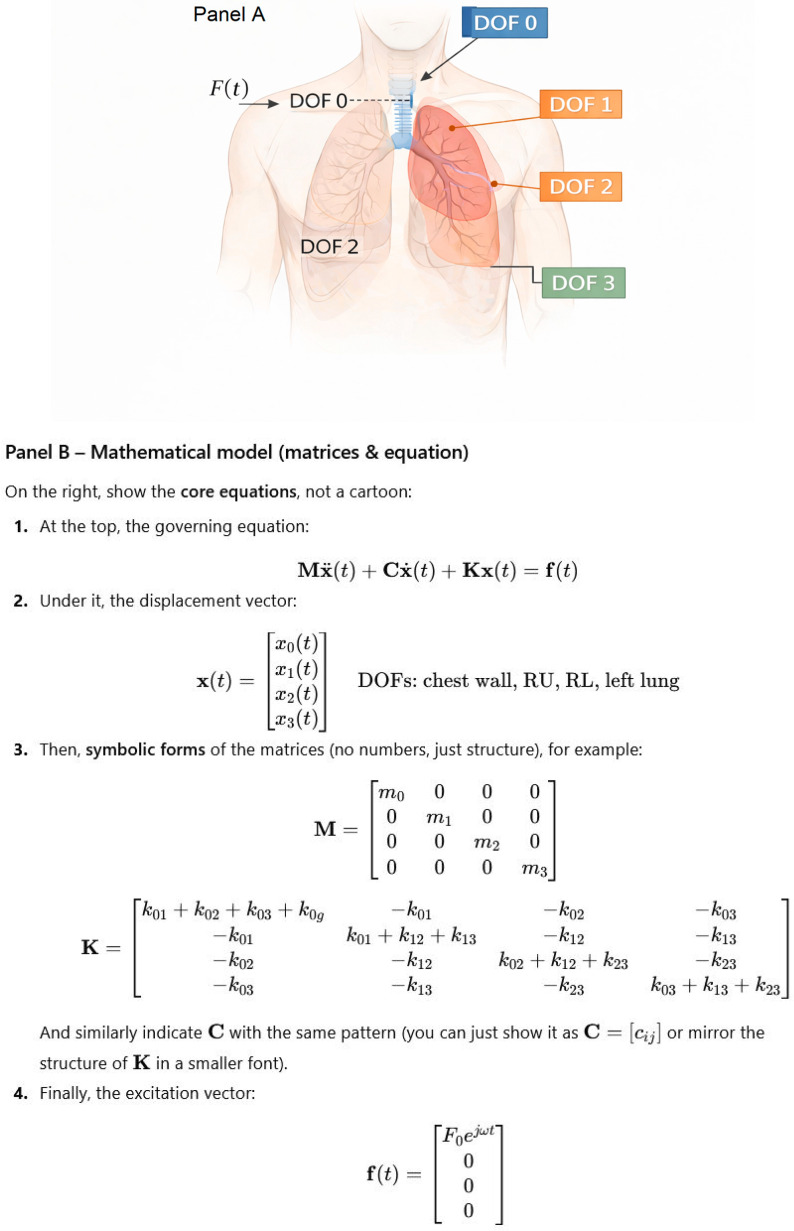
Anatomical representation and mathematical formulation of the mechanical lung–thorax model: (**A**) Schematic view of the thorax showing the four generalized degrees of freedom (DOFs) used in the model: chest wall (DOF 0), right upper lung region (DOF 1), right lower lung region (DOF 2), and left lung (DOF 3). External excitation is applied at the chest wall. (**B**) Mathematical representation of the model as a four-degree-of-freedom viscoelastic system.

The anatomical mapping of the generalized degrees of freedom and the corresponding mathematical formulation of the mechanical model is summarized in Figure 1.

(1) $$M\ddot{x}\left(t\right)+C\dot{x}\left(t\right)+Kx\left(t\right)=f(t)$$

where $x\left(t\right)={\left[x_{0},x_{1},x_{2},x_{3}\right]}^{T}$ and the mass, damping, and stiffness matrices **M**, **C**, **K** are defined in Section 2.1. The full mechanical coupling between compartments is encoded in these matrices; no additional structural assumptions are made beyond the lumped-parameter representation.

##### **Anatomical and Physical Justification of the Chosen Degrees of Freedom**

The degrees of freedom introduced in the present model do not correspond to sharply bounded anatomical structures in the strict histological sense. Instead, they represent effective mechanical compartments that aggregate regions of the lung and thorax with similar dynamic behavior under low-frequency external excitation.

This distinction is important. The purpose of the model is not to reproduce detailed lung anatomy, but to capture dominant modes of mechanical motion that are both physically meaningful and externally observable through the chest wall. Similar abstractions are widely used in respiratory mechanics, elastography, and vibration-based structural identification, where distributed continua are represented by a small number of generalized coordinates [12,15,19,20,21,22,23,28,35].

DOF 0 (Chest wall) represents the anterior thoracic surface, including the sternum, ribs, costal cartilage, and superficial tissues. This degree of freedom is anatomically well-defined and corresponds directly to the site of external excitation and measurement. In mechanical terms, it serves as the primary interface between the external actuator/sensor system and the internal lung structures. The chest wall is known to exhibit measurable compliance and inertia that significantly influence low-frequency respiratory mechanics.

DOF 1 (Right upper lung region) and DOF 2 (Right lower lung region) reflect the fact that the right lung is anatomically subdivided into upper and lower lobes that differ in geometry, ventilation, perfusion, and mechanical behavior. Gravity, airway branching, and pleural constraints lead to non-uniform regional mechanics even under passive conditions. Clinically, many lung pathologies—such as fibrosis, atelectasis, or localized consolidation—preferentially affect either upper or lower regions. Separating the right lung into two effective compartments therefore provides a physically motivated minimal resolution that allows regional mechanical heterogeneity to be represented and identified.

DOF 3 (Left lung, lumped) represents the left lung treated as a single effective mechanical compartment. While the left lung is anatomically divided into lobes, its overall volume and mediastinal constraints differ from those of the right lung. In the present proof-of-concept model, the left lung is intentionally lumped to limit the total number of degrees of freedom and preserve identifiability from external measurements. This choice reflects a common modeling strategy in which asymmetry is introduced only where it is expected to have the strongest diagnostic impact.

Importantly, each generalized displacement coordinate $x_{i}(t)$ should be interpreted as an effective modal coordinate, not as the physical displacement of a specific point or boundary within the lung. These coordinates describe the dominant collective motion of each compartment under small-amplitude excitation, analogous to low-order vibration modes in structural dynamics.

The boundaries between compartments shown schematically in Figure 1 are therefore conceptual rather than anatomical. They are not intended to imply sharp physical separations, but to indicate regions whose mechanical properties are aggregated into distinct parameters (mass, stiffness, and damping) within the lumped-parameter framework.

This level of abstraction is justified by the frequency range of interest. At low frequencies (below approximately 200 Hz), the lung–thorax system behaves predominantly as a coupled viscoelastic structure, and fine-scale wave propagation effects are strongly attenuated. A simple wavelength estimate further supports this modeling choice. The effective longitudinal wave speed in aerated lung tissue has been reported in the range of approximately 20–50 m/s, depending on inflation state and parenchymal stiffness. For excitation frequencies up to 150 Hz, the corresponding wavelengths are on the order of the range of 0.13–0.33 m, which are comparable to or exceed characteristic lung dimensions. Under such conditions, spatial phase variation within each lung region remains limited, and the dominant mechanical behavior is governed by collective viscoelastic motion rather than propagating wave phenomena. This justifies the use of a lumped-parameter representation for proof-of-concept system-identification within the selected frequency band. Under these conditions, regional mechanical differences manifest primarily through changes in global and semi-global dynamic responses, which can be captured by a small number of effective degrees of freedom.

Finally, it should be emphasized that the selected compartmentalization is not unique. The present four-degree-of-freedom configuration should therefore be interpreted as a minimal identifiable mechanical representation intended to establish proof-of-concept feasibility rather than a detailed anatomical reconstruction of lung mechanics. Alternative partitions (e.g., bilateral upper/lower regions, or additional lobar subdivisions) could be adopted as experimental resolution improves. The present four-degree-of-freedom configuration represents a minimal anatomically informed model that balances interpretability, identifiability, and diagnostic relevance, making it suitable for establishing the feasibility of mechanical system identification as a lung diagnostic paradigm.

#### 2.1.3. Mass Matrix

Inertial properties were represented by a diagonal mass matrix:

(2) $$M=\left[\begin{array}{cccc} m_{0} & 0 & 0 & 0\\ 0 & m_{1} & 0 & 0\\ 0 & 0 & m_{2} & 0\\ 0 & 0 & 0 & m_{3} \end{array}\right]$$

where:

- *m*_0_ denotes the effective inertia of the chest wall.
- *m*_1_, *m*_2_, *m*_3_ denote effective inertial masses of the corresponding lung regions.

Each mass parameter represents the combined inertia of tissue, entrained air, and blood volume within the compartment and should be interpreted as an effective dynamic quantity rather than anatomical mass.

#### 2.1.4. Stiffness Matrix and Elastic Coupling

Elastic restoring forces were modeled using linear springs representing tissue elasticity, pleural coupling, air compressibility, and thoracic constraints.

Elastic connections were defined between the following compartment pairs (depicted in Table 2).

The stiffness matrix **K** was assembled such that each spring between degrees of freedom *i* and *j* contributed:

(3) $$K_{ii}+=k_{ij},K_{jj}+=k_{ij},K_{ij}-=k_{ij},K_{ji}-=k_{ij},$$

This construction ensures global force balance and symmetric coupling.

Pathological changes were modeled as localized perturbations of stiffness values:

- Fibrosis was represented by increased stiffness in affected regions.
- Emphysema by reduced stiffness.
- Focal lesions by combined stiffness and mass changes.

#### 2.1.5. Damping Matrix

Viscous dissipation was represented by linear dampers arranged with the same topological structure as the elastic springs. Damping accounts for:

- Tissue viscosity.
- Air friction.
- Microstructural hysteresis.
- Blood flow-related dissipation.
- The damping matrix **C** was assembled analogously to the stiffness matrix:

(4) $$C_{ii}+=c_{ij},C_{ij}-=c_{ij}$$

Damping ensures finite resonance amplitudes and realistic phase behavior in the frequency response.

#### 2.1.6. Governing Equations of Motion

The dynamics of the system are governed by the linear second-order matrix equation:

(5) $$M\ddot{x}\left(t\right)+C\dot{x}\left(t\right)+Kx\left(t\right)=f(t)$$

where $x\left(t\right)={\left[x_{0},x_{1},x_{2},x_{3}\right]}^{T}$ and f(t) is the external excitation force vector.

#### 2.1.7. External Excitation

External excitation was applied exclusively at the chest wall degree of freedom to emulate non-invasive actuation:

(6) $$f\left(t\right)=\left[\begin{array}{c} F_{0}e^{j\omega t}\\ 0\\ 0\\ 0 \end{array}\right]$$

This formulation represents gentle mechanical or acoustic excitation transmitted through the chest surface, without any internal forcing.

#### 2.1.8. Frequency-Domain Formulation

Assuming steady-state harmonic response of the form:

(7) $$x\left(t\right)=X(\omega )e^{j\omega t}$$

the governing equations reduce to:

(8) $$\left(-\omega^{2}M+j\omega C+K\right)X=F$$

For each excitation frequency ω, this complex linear system was solved to obtain the steady-state response vector X(ω).

#### 2.1.9. Frequency Response Functions

The system frequency response function (FRF) is defined as:

(9) $$H\left(\omega \right)={\left(-\omega^{2}M+j\omega C+K\right)}^{-1}$$

Measured quantities correspond to the magnitude and phase of individual components $X_{i}(\omega )$ which may represent surface displacement, velocity, acceleration, or acoustically coupled signals depending on sensor modality.

#### 2.1.10. Inverse Analysis and Parameter Identification

Changes in internal mechanical properties were inferred by comparing measured frequency response functions with model predictions. Identifiability is enabled by:

- Multi-frequency excitation.
- Global mode coupling.
- Non-degenerate parameter sensitivity.

Inverse estimation focused on recovering localized stiffness perturbations associated with simulated pathological conditions.

#### 2.1.11. Model Limitations

The present model neglects nonlinear tissue behavior, airflow turbulence, and active muscle contraction and does not attempt anatomical geometric fidelity. Its purpose is to establish physical identifiability and feasibility, not to provide a complete physiological description.

#### 2.1.12. Parameter Perturbations and Pathological Configurations

Pathological lung conditions were simulated as localized perturbations of mechanical parameters relative to a healthy reference configuration. Perturbations were introduced in a controlled manner to isolate the effect of regional mechanical changes on the system response.

Three configurations were considered:

- Healthy reference state, characterized by baseline mass, stiffness, and damping parameters.
- Right upper lung pathology, implemented as a localized increase in stiffness parameters associated with the right upper lung compartment.
- Right lower lung pathology, implemented as a localized increase in stiffness parameters associated with the right lower lung compartment.

All perturbations were applied exclusively to selected stiffness terms within the matrix **K**, while mass and damping parameters were held fixed. This approach reflects pathological conditions such as fibrosis, where increased tissue stiffness is the dominant mechanical alteration. Perturbation magnitudes were chosen to produce measurable yet physiologically plausible changes in the frequency response without violating the linear regime.

We emphasize, however, that real pulmonary pathologies are generally multi-parametric and may involve concurrent alterations in effective damping, inertial loading, and inter-compartmental coupling in addition to stiffness changes. The present study isolates stiffness perturbations intentionally in order to establish identifiability in a controlled proof-of-concept setting.

#### 2.1.13. Noise Model

To assess robustness under realistic measurement conditions, additive noise was introduced into the simulated frequency response data. Noise was modeled as additive zero-mean Gaussian perturbations applied independently to the magnitude of the simulated frequency response at each frequency point. The noise was introduced after the generation of simulated responses and prior to inverse fitting and classification analyses. The noise amplitude was scaled relative to the response magnitude to emulate moderate experimental variability while preserving diagnostically relevant response features. Phase information and underlying model parameters were left unperturbed in order to isolate the effect of measurement noise on observable response amplitudes. This noise model was applied consistently in both inverse fitting and classification analyses.

#### 2.1.14. Inverse Fitting Procedure

An inverse estimation of internal mechanical parameters was performed by minimizing the discrepancy between simulated “measured” frequency response data and model-predicted responses. The objective function was defined as the sum of squared differences between response magnitudes over the analyzed frequency range.

Optimization was restricted to a subset of stiffness parameters corresponding to chest–lung and inter-lung couplings, while mass and damping parameters were fixed at baseline values. This restriction reflects a diagnostic scenario in which changes in tissue stiffness are of primary interest.

Nonlinear least-squares optimization was employed to recover stiffness parameters by minimizing the squared discrepancy between simulated measurement data and model-predicted frequency response magnitudes over the analyzed frequency band. Initial parameter values were set to the healthy reference configuration used in the forward simulations. Parameter estimates were allowed to vary within physically plausible ranges around the baseline configuration to prevent non-physical solutions.

Convergence was assessed based on the stability of recovered parameter values and reduction of the objective function between successive iterations, together with agreement between predicted and measured frequency responses across the analyzed frequency band. Robustness of parameter recovery under moderate noise levels and the simultaneous estimation of multiple stiffness parameters were further evaluated through the sensitivity and Fisher-information analyses presented in Section 3.3.

#### 2.1.15. Classification Pipeline

To assess diagnostic discriminability, frequency response data from multiple simulated realizations were processed through a classification pipeline. Feature vectors were constructed from the magnitude of the frequency response over the selected frequency range. Dimensionality reduction was applied prior to classification to mitigate redundancy and noise sensitivity.

For the classification experiment, synthetic datasets were generated for three conditions (healthy lung, right upper-lobe fibrosis, and right lower-lobe fibrosis), with 30 simulated realizations per class (total *n* = 90). Each realization consisted of the magnitude of the frequency response measured at four degrees of freedom over 300 frequency points between 5 and 150 Hz. Feature vectors were flattened and reduced using principal component analysis (PCA) to six principal components.

The resulting dataset was randomly divided into training (70%, *n* = 63) and test (30%, *n* = 27) subsets using stratified sampling to preserve class balance. A multinomial logistic regression classifier (max_iter = 1000) was trained on the training set and evaluated on the held-out test set. Classification performance was quantified using precision, recall, and F1-score metrics.

#### 2.1.16. Summary of Methodological Workflow

The complete methodological workflow comprised:

1. Construction of a multi-degree-of-freedom mechanical lung–thorax model.
2. Simulation of frequency-domain responses under controlled external excitation.
3. Introduction of localized stiffness perturbations to represent pathological conditions.
4. Addition of measurement noise to simulated response data.
5. Inverse recovery of internal mechanical parameters from external measurements.
6. Classification of pathological configurations based on frequency response features.

### 2.2. External Excitation and Signal Acquisition

#### 2.2.1. Input Excitation

Mechanical excitation of the lung–thorax system was modeled as a controlled, low-amplitude external input applied at the chest wall. In practice, such excitation may be delivered using non-invasive surface actuation, including gentle mechanical vibration or low-intensity acoustic stimulation, applied over the sternum or anterior rib cage.

The input was represented mathematically as a harmonic force applied exclusively to the chest wall degree of freedom (DOF 0):

(10) $$f\left(t\right)=\left[\begin{array}{c} F_{0}e^{j\omega t}\\ 0\\ 0\\ 0 \end{array}\right]$$

where $F_{0}$ denotes the excitation amplitude and *ω* the angular frequency. Excitation amplitudes were assumed to remain within a small-signal regime to ensure linear system behavior and subject comfort.

To characterize the system over a frequency range, excitation frequencies were swept across the low-frequency band of interest, and steady-state responses were computed at each frequency. This approach enables construction of frequency response functions without requiring large transient inputs.

#### 2.2.2. Measurement and Signal Acquisition

System response was assumed to be measured non-invasively at the chest surface. Depending on the sensing modality, measured signals may correspond to surface displacement, velocity, acceleration, or acoustically coupled pressure fluctuations. In the present study, measurements were represented abstractly as frequency-domain response components *X_i_*(*ω*), without restricting the analysis to a specific sensor technology. In practical implementations, accelerometers, piezoelectric acoustic sensors, or ultrasound probes operated in a motion-tracking mode could provide the required surface response measurements [26,27,29,36,37].

For each excitation frequency, the steady-state response was extracted after transient effects had decayed. The magnitude of the response was used as the primary observable for inverse analysis and classification, reflecting realistic measurement scenarios in which phase information may be less robust or more difficult to acquire.

**Figure 2:**
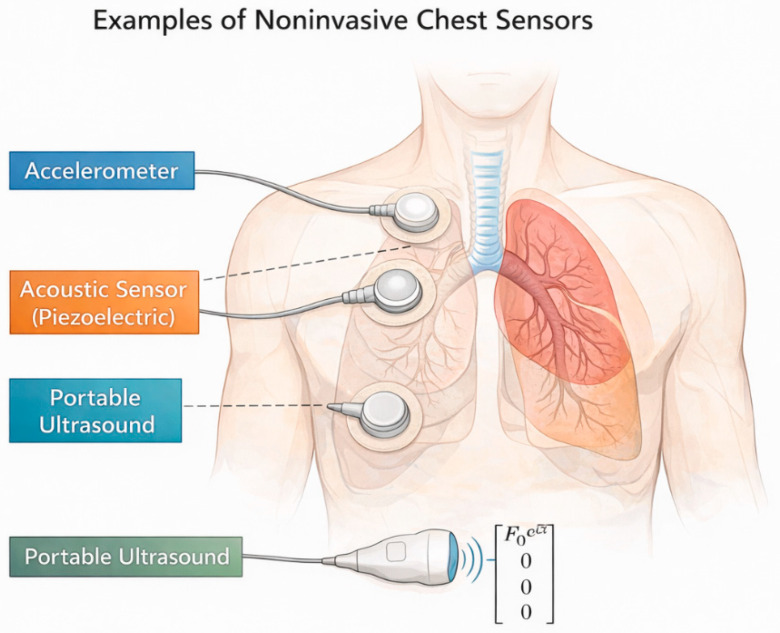
Examples of non-invasive sensor modalities for external excitation and response measurements. Illustration showing representative non-invasive sensors that may be used to excite and/or measure the mechanical response of the lung–thorax system at the chest wall. Shown examples include a surface accelerometer, a piezoelectric acoustic sensor, and a portable ultrasound probe positioned over the anterior thorax. Sensors are placed externally and do not require internal instrumentation. The proposed diagnostic framework is independent of the specific sensing modality and relies on measured surface responses to controlled external excitation.

Examples of non-invasive sensor modalities and their placement on the chest wall are illustrated in Figure 2.

#### 2.2.3. Signal Processing and Frequency Response Estimation

Measured time-domain signals were assumed to be processed using standard spectral analysis techniques to obtain frequency-domain representations. For harmonic excitation, the frequency response at each degree of freedom was computed as the ratio between the measured response amplitude and the known excitation amplitude at the corresponding frequency.

This procedure yields frequency response functions of the form:

(11) $$X_{i}\left(\omega \right)=H_{i}\left(\omega \right)F_{0}$$

where $H_{i}\left(\omega \right)$ denotes the transfer function between the applied chest wall excitation and the measured response at compartment *i*.

To ensure consistency across simulations, frequency response magnitudes were sampled uniformly over the selected frequency range and assembled into feature vectors for subsequent inverse fitting and classification analyses.

#### 2.2.4. Safety and Physiological Considerations

All excitation and measurement assumptions were restricted to low-amplitude, low-frequency conditions consistent with non-invasive mechanical testing of the chest wall. The proposed framework does not require internal instrumentation, airflow manipulation, or breath-holding maneuvers, and excitation levels were chosen to remain well below thresholds associated with discomfort or tissue stress.

#### 2.2.5. Role Within the Diagnostic Framework

Within the proposed diagnostic model, external excitation and signal acquisition serve solely to probe the passive mechanical properties of the lung–thorax system. Diagnostic inference is performed by interpreting the resulting frequency response functions through a physically grounded model, rather than relying on raw signal patterns alone.

#### 2.2.6. End of Methods

This modeling framework enables a non-invasive inference of regional lung mechanical properties by treating the lung–thorax system as an identifiable viscoelastic dynamical system under controlled external excitation.

## 3. Results

### 3.1. Frequency Response of the Mechanical Lung Model

The frequency response of the four-degree-of-freedom lung–thorax model was evaluated under harmonic excitation applied at the chest wall. Steady-state responses were computed over the low-frequency mechanical range relevant to the model assumptions.

**Figure 3:**
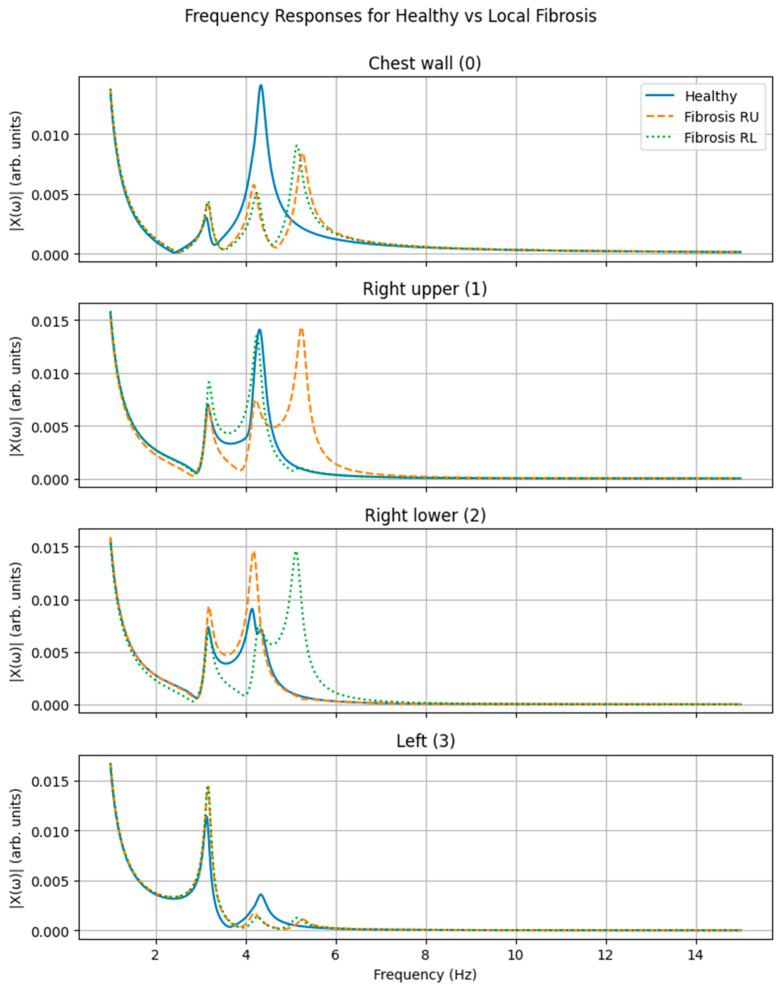
Frequency response functions of the mechanical lung–thorax model under localized stiffness perturbations. Magnitude of the steady-state frequency response functions ∣*X_i_*(*ω*)∣ for each degree of freedom of the four-degree-of-freedom lung–thorax model under harmonic excitation applied at the chest wall (DOF 0). Responses are shown for a healthy reference configuration, localized stiffness increase in the right upper lung region (fibrosis RU), and localized stiffness increase in the right lower lung region (fibrosis RL). Separate panels correspond to the chest wall (DOF 0), right upper lung region (DOF 1), right lower lung region (DOF 2), and left lung (DOF 3). All responses are plotted on identical frequency and amplitude scales.

Figure 3 shows the magnitude of the frequency response functions (FRFs) for all degrees of freedom—chest wall, right upper lung region, right lower lung region, and left lung—for three simulated configurations: a healthy reference state, localized stiffness increases in the right upper lung region, and localized stiffness increase in the right lower lung region.

In the healthy configuration, the system exhibits smooth and continuous frequency-dependent responses across all compartments, with multiple resonance peaks reflecting the coupled dynamics of the chest wall and lung regions. When stiffness was selectively increased in the right upper lung region, distinct modifications were observed in the frequency response of the corresponding compartment, including shifts in resonance frequency and changes in response amplitude. These alterations were most pronounced in the right upper lung response but were also detectable in the chest wall response, despite excitation and measurement being applied externally.

Similarly, localized stiffening of the right lower lung region produced characteristic changes in the frequency response of the right lower compartment. The resulting response pattern differed from that observed for right upper lung stiffening, with distinct resonance shifts and amplitude redistribution. Responses of the remaining compartments exhibited comparatively smaller changes, preserving a different frequency signature.

Overall, the frequency-domain signatures associated with right upper and right lower stiffness perturbations were clearly distinguishable from one another and from the healthy reference configuration, indicating sensitivity of the global frequency response to localized mechanical alterations.

### 3.2. Inverse Recovery of Regional Stiffness Parameters

To assess the identifiability of internal mechanical parameters, inverse fitting was performed using simulated frequency response magnitude data corrupted with measurement noise. The inverse problem was formulated to recover selected chest–lung stiffness parameters while all other model parameters were held fixed.

**Figure 4:**
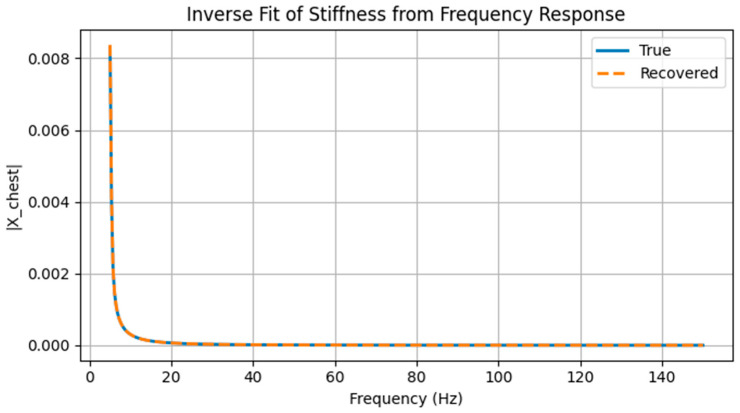
Inverse recovery of chest–lung stiffness from frequency response data. Magnitude of the chest wall frequency response ∣*X_chest_*(*ω*)∣ for a synthetic “true” configuration (solid line) and for the model using stiffness parameters recovered by nonlinear least-squares fitting (dashed line). The near overlap between curves indicates accurate recovery of internal stiffness parameters from noisy external measurements.

Figure 4 compares the chest wall frequency response magnitude obtained from simulated noisy measurements with the response predicted by the model using stiffness parameters recovered through nonlinear least-squares optimization. The recovered model closely reproduces the measured response across the analyzed frequency range, capturing both the overall amplitude and the frequency-dependent structure of the response.

The true stiffness values used to generate the synthetic measurements were *k*_01_ = 240 and *k*_02_ = 154 (arbitrary units), corresponding to chest–right-upper and chest–right-lower couplings, respectively. Inverse fitting yielded recovered values of *k*_01_ = 239.5 and *k*_02_ = 159.5. The close agreement between true and recovered parameters demonstrates that localized stiffness perturbations can be inferred from external frequency response measurements, even in the presence of moderate noise.

Although inverse estimation was performed using nonlinear least-squares optimization without explicit regularization, the problem remained well-conditioned within the examined parameter subspace, as confirmed by the sensitivity and Fisher-information analysis presented in Section 3.3. In exploratory tests, the inclusion of a Tikhonov regularization term yielded parameter estimates within a few percent of the unregularized solution under moderate noise levels, indicating numerical stability for the simplified synthetic scenario. In practical experimental settings, where model mismatch and measurement uncertainty may be greater, regularized or Bayesian estimation strategies would likely be required to ensure stable parameter recovery.

### 3.3. Sensitivity and Identifiability of Regional Stiffness Parameters

To assess whether regional chest–lung stiffness parameters are structurally identifiable from externally measured dynamics, a finite-difference sensitivity analysis was performed around the healthy baseline configuration. The chest–right-upper stiffness *k*_01_ and chest–right-lower stiffness *k*_02_ were perturbed individually by 1%, and the resulting changes in the multi-output frequency response magnitude ∣*X_i_*(*f*)∣ were evaluated across the low-frequency band.

Sensitivities were expressed in dimensionless relative form as

(12) $$\frac{\partial \ln\left|X_{i}(f)\right|}{\partial \ln k_{j}}$$

which quantify the percentage change in response amplitude induced by a percentage change in stiffness.

**Figure 5:**
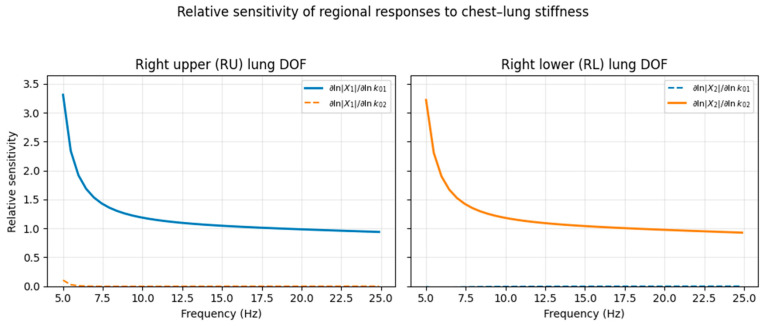
Relative sensitivity of RU and RL lung responses to regional chest–lung stiffness. Dimensionless sensitivities $\partial \ln\left|X_{i}(f)\right|/\partial \ln k_{j}$ for the right upper (RU) and right lower (RL) lung degrees of freedom over the range of 5–25 Hz. The RU response is dominated by *k*_01_ (chest–right upper coupling), while the RL response is dominated by *k*_02_ (chest–right lower coupling), confirming directional separation and local identifiability of regional stiffness parameters.

Figure 5 shows the relative sensitivities for the right upper (RU) and right lower (RL) lung degrees of freedom over the 5–25 Hz band. The RU response exhibits strong sensitivity to *k*_01_ with negligible dependence on *k*_02_, whereas the RL response displays the opposite pattern. This directional dominance persists across the low-frequency range and indicates that the two regional stiffness parameters modulate distinct dynamical features of the system response.

To quantify identifiability more formally, sensitivities from all outputs and frequencies were stacked to form a global Jacobian matrix. The associated Fisher-information matrix had two strictly positive eigenvalues (λ_1_ = 1.75 × 10^−9^, λ_2_ = 8.91 × 10^−10^) and a low condition number (*κ*(*F*) ≈ 1.96), with modest correlation between sensitivity directions (ρ ≈ 0.29). These results demonstrate that the regional stiffness parameters are locally identifiable in the proposed multi-output configuration.

### 3.4. Discrimination of Pathological Conditions Using Response Features

To evaluate whether the observed frequency response differences are sufficient for diagnostic discrimination, frequency response data from multiple simulated realizations were analyzed using a classification pipeline. For each realization, frequency response magnitudes from all degrees of freedom were assembled into feature vectors and reduced in dimensionality prior to classification.

**Figure 6:**
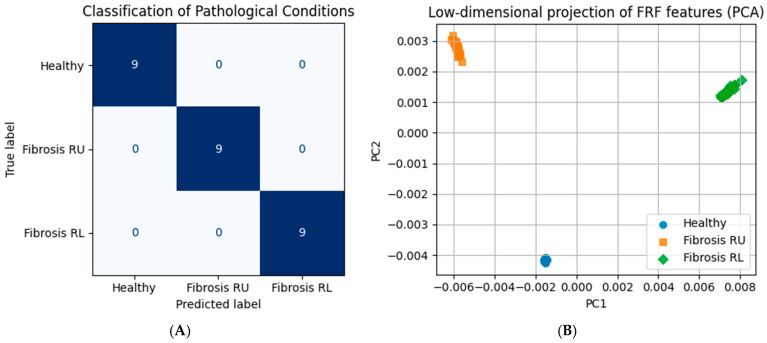
Discrimination of pathological configurations using frequency response features. (**A**) Confusion matrix summarizing classification performance for healthy lung, localized stiffness increase in the right upper lung region (fibrosis RU), and localized stiffness increase in the right lower lung region (fibrosis RL). (**B**) Projection of frequency response magnitude features onto the first two principal components (PC1 and PC2), showing a clear separation of the three configurations in a low-dimensional feature space.

Figure 6A presents the confusion matrix summarizing classification performance for three simulated conditions: healthy lung, localized stiffness increase in the right upper lung region, and localized stiffness increase in the right lower lung region. Under the controlled simulation conditions considered here, all test samples were correctly classified, indicating complete separability of the three configurations in the chosen feature space.

Quantitative classification metrics are reported in Table 3. Precision, recall, and F1-score were equal to 1.00 for all three classes.

To visualize the structure of the feature space independently of the classifier, the same frequency response features were projected onto the first two principal components. Figure 6B shows the resulting low-dimensional representation, where each point corresponds to a single simulated realization. The three configurations form well-separated clusters in the PC1–PC2 plane, indicating that discriminative information is preserved in the dominant variance of the frequency response data.

To assess robustness beyond the idealized simulation conditions shown here, additional analyses were performed to quantify the effects of measurement noise and inter-subject mechanical variability on classification performance. These Supplementary Experiments demonstrate that while variability reduces visual separability in low-dimensional projections, supervised classification using full frequency response features remains robust and well above chance levels across a wide range of noise and parameter perturbations (Supplementary Figures S1–S3).

It should be emphasized that these results demonstrate theoretical discriminability under idealized modeling assumptions rather than expected diagnostic performance in experimental or clinical settings.

More challenging scenarios, including milder perturbations, mixed pathology patterns, and stronger inter-subject variability, will be important in future work to establish practical classification limits.

### 3.5. Summary of Key Results

Across all analyses, three principal results emerge. First, localized mechanical alterations produce distinct and reproducible signatures in the global frequency response of the lung–thorax system. Second, internal stiffness parameters associated with regional pathology can be accurately recovered through inverse analysis of externally measured frequency responses, even in the presence of measurement noise. Third, frequency response features enable reliable discrimination between different regional pathological configurations. Together, these results establish the feasibility of mechanical system identification as a basis for a non-invasive assessment of regional lung mechanics, with robustness to measurement noise and physiologically plausible parameter variability demonstrated in Supplementary Analyses.

## 4. Discussion

### 4.1. From Simulation to Diagnostic Model

In this study, we introduced and evaluated a mechanical system-identification framework for lung assessment, in which internal mechanical properties are inferred from externally measured frequency responses. While the results presented here are based on simulated data, the significance of the work lies not in numerical performance alone, but in establishing the conceptual feasibility of a diagnostic model for the lung grounded in mechanical system identification.

Conventional lung diagnostics are largely divided between structural imaging modalities, which provide spatially resolved but static information, and global functional tests, which provide dynamic but spatially averaged measures. The approach presented here occupies a distinct conceptual space: the lung–thorax system is treated as a physical dynamical system, and diagnosis is performed by identifying internal mechanical parameters from its externally observable response. In this framework, pathology is not detected as an image or a scalar index, but as a change in the system’s dynamical signature.

The results demonstrate that localized alterations in lung stiffness generate distinct and recoverable changes in the frequency response of the system, even when excitation and measurement are applied exclusively at the chest wall. This demonstrates the theoretical feasibility of detecting regional mechanical changes from externally measured responses without direct internal access or imaging.

### 4.2. Relation to Existing Lung Diagnostic Methods

Current approaches to lung diagnostics can be broadly categorized into structural imaging techniques, such as chest radiography and computed tomography, and functional tests, such as spirometry and oscillometry. Structural imaging provides spatially resolved information on lung morphology, including consolidation, fibrosis patterns, or emphysema distribution, but it is inherently static and does not directly quantify regional mechanical behavior. Global pulmonary function tests, by contrast, quantify airflow and volume changes, yielding scalar indices such as FEV_1_ or total lung capacity that reflect overall respiratory performance but do not resolve regional heterogeneity.

Mechanical assessment of lung function has also been pursued using oscillatory techniques, most prominently the forced oscillation technique (FOT) and impulse oscillometry, which measure respiratory system impedance at the airway opening. These methods typically summarize the response in terms of resistance and reactance at a limited number of frequencies and are often interpreted using low-order or compartment-averaged models. While they provide valuable information about global mechanics and have demonstrated clinical utility, their ability to localize mechanical abnormalities to specific lung regions is limited.

The approach proposed in this work differs fundamentally from these existing methods. Rather than imaging anatomy or measuring airflow-based indices, the lung–thorax system is explicitly treated as a coupled multi-degree-of-freedom dynamical system, and diagnostic information is extracted from its low-frequency frequency response to external excitation. Internal mechanical properties, such as regional stiffness and coupling strength, are inferred through system identification using the full frequency response function, rather than through impedance summaries or image-derived features. In this framework, pathology manifests as a change in the system’s dynamical signature, not as a radiological pattern or a single global index.

The framework is also distinct from ultrasound imaging and ultrasound elastography. Conventional lung ultrasound relies on high-frequency wave propagation and image or artifact formation at air–tissue interfaces, whereas elastography seeks to reconstruct local stiffness from spatial strain patterns or shear wave propagation. Both approaches are challenged by the highly aerated and heterogeneous nature of lung tissue. In contrast, the present method operates in a low-frequency regime, does not require wave penetration into the parenchyma, and does not attempt to image tissue directly. Ultrasound, if employed, serves only as an external motion sensor at the chest wall, providing input to a mechanical system-identification model rather than acting as an imaging modality.

By focusing on mechanical dynamics rather than structure or airflow alone, the proposed framework introduces a complementary diagnostic dimension. It aims to recover regional mechanical properties from non-invasive surface measurements, thereby bridging the gap between global functional tests and structurally detailed but mechanically indirect imaging modalities.

#### **Distinction from Forced Oscillation and Impedance-Based Techniques**

Forced oscillation techniques and impulse oscillometry have demonstrated clinical utility by characterizing respiratory system impedance under small-amplitude oscillatory excitation. However, these methods typically apply excitation at the airway opening and interpret the response using scalar resistance and reactance measures or low-order compartment models that represent the lung as a largely homogeneous system. The framework presented here differs in both excitation strategy and diagnostic objective. Excitation is applied externally at the chest wall, and the lung–thorax system is modeled as a coupled multi-degree-of-freedom dynamical system. Diagnostic inference is performed by identifying internal mechanical parameters—such as regional stiffness—from the full frequency response function, rather than by summarizing impedance at the airway opening. As a result, the proposed approach is not an extension of oscillometry, but a shift toward vibration-based system identification analogous to methods used in structural health monitoring, adapted here for a non-invasive assessment of lung mechanics.

### 4.3. Physical Origin of Diagnostic Sensitivity

The ability to infer regional pathology from external measurements arises from fundamental properties of coupled dynamical systems. In such systems, local changes in stiffness or damping alter not only local responses but also global modal structure, leading to shifts in resonance frequencies, changes in mode shapes, and a redistribution of response energy across degrees of freedom.

In the lung–thorax system, mechanical coupling through the chest wall, pleura, and mediastinum ensures that regional alterations propagate to the surface. The frequency-domain analysis presented here shows that these effects are detectable even under small-amplitude excitation and in the presence of measurement noise. This is consistent with analogous approaches in structural health monitoring, where defects in inaccessible regions of a structure are identified through vibration-based analysis [26,27,36,37].

The clear separation of pathological configurations in the low-dimensional principal component space further indicates that diagnostic information is encoded in the dominant variance of the response, rather than in subtle or noise-sensitive features. This suggests that the approach may be robust to measurement variability and sensor limitations.

### 4.4. Identifiability and Model Order Considerations

The mechanical model employed in this study represents the lung–thorax system using a small number of effective degrees of freedom. This choice reflects a balance between anatomical relevance and the practical limits of inverse parameter identification from externally measured responses. In general, the number of internal mechanical parameters that can be reliably identified is constrained by the information content of the measured frequency response, which depends on excitation bandwidth, signal-to-noise ratio, and the strength of mechanical coupling between compartments.

The four-degree-of-freedom configuration adopted here should therefore be interpreted as a minimal identifiable model rather than a detailed anatomical decomposition. The chest wall degree of freedom provides a well-defined interface for external excitation and measurement, while the subdivision of the right lung into upper and lower effective compartments enables representation of clinically meaningful regional heterogeneity without excessive parameterization. The left lung is intentionally lumped to limit model complexity and preserve robustness of the inverse problem under realistic noise conditions.

Importantly, the generalized coordinates of the model correspond to effective modal motions rather than discrete anatomical structures. At the low frequencies considered in this study, the lung–thorax system behaves predominantly as a coupled viscoelastic structure, and its dynamics are governed by a small number of dominant modes. Under these conditions, localized changes in mechanical properties influence the global frequency response through shifts in resonance frequencies, mode shapes, and energy distribution across degrees of freedom, enabling regional inference even with a low-order representation.

As experimental resolution and measurement fidelity improve, higher-order models incorporating additional regional subdivisions may become identifiable. In practice, appropriate model order would be determined using standard system-identification criteria, including parameter sensitivity, stability of recovered estimates, and consistency of model fits across frequency. The present results demonstrate that even a low-order model suffices to recover localized stiffness alterations and discriminate between regional pathological configurations, thereby establishing a lower bound on the mechanical resolution achievable through non-invasive system identification of the lung–thorax system.

The explicit sensitivity and Fisher-information analysis presented in Section 3.3 confirms that the regional stiffness parameters are locally identifiable from multi-output frequency responses and that the proposed model is not over-parameterized in the stiffness subspace relevant to simulated pathology.

An important next step will be to evaluate identifiability under simultaneous perturbations of stiffness, damping, and coupling parameters, where parameter cross-correlation and local minima may become more significant.

### 4.5. Implications for Pulmonary Rehabilitation

Pulmonary rehabilitation aims to improve exercise capacity, symptoms, and quality of life by targeting the integrated function of the lung–thorax system, peripheral muscles, and cardiovascular responses. In current clinical practice, enrollment and progression through rehabilitation programs are guided largely by global indices such as FEV_1_, diffusion capacity, exercise tests, and symptom scores. Although these measures are highly relevant, they do not explicitly quantify regional mechanical abnormalities or their distribution across the lung and chest wall. As a result, patients with distinct mechanical phenotypes may receive similar rehabilitation prescriptions, and heterogeneity in response to standardized programs is common.

Within this context, the present mechanical system-identification framework suggests a complementary route toward more individualized pulmonary rehabilitation. By recovering physically interpretable parameters, such as regional stiffness and inter-compartment coupling from non-invasive surface measurements, the model provides a structured representation of mechanical heterogeneity that could be used to stratify patients before rehabilitation, identify those with asymmetrical or highly localized stiffness patterns, and define mechanistic targets for intervention. In principle, longitudinal application of the same identification procedure could also be used to track changes in regional mechanics over time, offering an objective mechanical correlation of rehabilitation-induced adaptation alongside traditional functional outcomes.

Importantly, the current study does not evaluate pulmonary rehabilitation directly and should not be interpreted as evidence of rehabilitation efficacy. Rather, it establishes that regional mechanical alterations are, in principle, identifiable from external dynamics and that these identifiability properties create a natural bridge between mechanical modeling and rehabilitation planning. Future work will be required to link specific mechanical phenotypes and parameter trajectories to clinically observed rehabilitation responses, and to determine whether system-identification-based metrics can improve patient selection, tailoring of exercise prescriptions, or monitoring of long-term outcomes compared with conventional assessment alone.

### 4.6. Diagnostic Interpretation and Clinical Relevance

Within the proposed framework, diagnosis is naturally framed as parameter identification rather than pattern recognition. Regional stiffness estimates provide physically interpretable quantities that can be related to known pathological processes, such as fibrosis or emphysematous tissue destruction. Unlike purely data-driven classifiers, this parameter-based representation supports mechanistic interpretation and longitudinal tracking. Accordingly, the perfect classification observed in the present simulations should be understood as demonstrating the existence of diagnostically separable mechanical signatures under idealized conditions, rather than as a prediction of achievable classification accuracy in clinical practice. In particular, future studies should test the ability to detect subtle mechanical abnormalities and to distinguish mixed regional patterns under higher noise and model-mismatch conditions.

Moreover, the non-invasive nature of excitation and measurement suggests potential applicability in populations for whom conventional imaging may be impractical or undesirable, such as pediatric or critically ill patients. While the present study does not address clinical implementation directly, it establishes the physical plausibility of inferring regional lung mechanics from external measurements alone.

### 4.7. Limitations of the Present Study

Several limitations should be acknowledged. The mechanical model employed here is intentionally simplified, relying on a lumped-parameter representation and linearized dynamics. While appropriate for a proof-of-concept investigation, this abstraction does not capture the full anatomical complexity or nonlinear behavior of the lung. In addition, the chosen model order reflects a trade-off between spatial resolution and identifiability from surface-only measurements, and should be viewed as a minimal representation rather than a definitive anatomical partition. Accordingly, the present framework should be viewed as a proof-of-concept model applicable to passive low-frequency mechanical interrogation, rather than a complete physiological description of respiration under all operating conditions.

Additionally, airflow dynamics, active muscle contraction, and posture-dependent effects were neglected. These factors will need to be addressed in future extensions of the model. The noise model used in this study was also simplified, with additive uncorrelated Gaussian noise applied only to the magnitude of the frequency response. Phase noise, sensor bias, and correlated measurement artifacts were not explicitly modeled, representing a best-case experimental scenario. Incorporating more realistic noise characteristics and experimental uncertainties will be an important focus of future work.

Finally, all results were obtained using simulated data, and experimental validation remains an essential next step. These limitations, however, do not undermine the central contribution of the work, which is the establishment of identifiability and diagnostic feasibility within a physically grounded framework.

Future work will address these limitations through staged experimental validation, beginning with physical thoracic phantoms with tunable viscoelastic properties, followed by pilot measurements in human volunteers under controlled low-amplitude excitation. These studies will allow the calibration of the mechanical model, evaluation of realistic measurement noise, and assessment of parameter recovery under physiological variability.

Future model extensions should therefore incorporate combined perturbations in stiffness, damping, and coupling to better represent mixed or evolving pathological phenotypes.

### 4.8. Path Toward Experimental and Clinical Validation

Because all the results in the present study are simulation-based, experimental validation represents the essential next step toward translation.

The modeling framework presented here is well-suited for staged experimental validation. As an initial step, physical thoracic phantoms with tunable stiffness and damping properties could be constructed using layered viscoelastic materials and air-filled compartments to emulate regional lung mechanics. Such phantoms would enable a controlled evaluation of parameter recovery accuracy, sensitivity to stiffness contrast, excitation bandwidth selection, and sensor placement strategies under reproducible conditions.

From an instrumentation perspective, the required excitation amplitudes are modest. Order-of-magnitude estimates indicate that chest wall displacements on the scale of tens to hundreds of micrometers are sufficient to generate measurable frequency response signatures within the 5–150 Hz band range while remaining well within safe and comfortable limits for passive thoracic vibration. Broadband excitation across this frequency range could be delivered using a lightweight electromechanical shaker, voice-coil actuator, or instrumented impulse hammer applied over the sternum or anterior rib cage.

Surface response measurements could be obtained using contact accelerometers, piezoelectric acoustic sensors, or motion-tracking ultrasound probes with bandwidths exceeding 200 Hz. Commercial accelerometers with sensitivities in the range of 10–100 mV/g are capable of detecting sub-millimeter thoracic vibrations, suggesting that signal-to-noise ratios comparable to those assumed in the present simulations are achievable under controlled conditions. Because the proposed framework relies primarily on the structure of the frequency response rather than absolute displacement magnitude, moderate variability in sensor placement or baseline amplitude is not expected to compromise parameter identifiability.

Following phantom validation, pilot studies in human subjects under carefully controlled, low-amplitude excitation could be conducted using existing vibration or oscillatory technologies. Importantly, the framework does not require new imaging hardware but instead reframes externally measured mechanical response data within a physically grounded system-identification paradigm. This staged pathway—from controlled phantoms to feasibility testing in volunteers—provides a realistic route toward eventual clinical translation.

Future experimental studies should also compare alternative excitation protocols, including harmonic sweeps, broadband random excitation, and impulse-like inputs, in order to determine which waveform provides the best trade-off between identifiability, robustness, and ease of bedside implementation.

### 4.9. Implications and Outlook

By reframing lung diagnostics as a problem of mechanical system identification, this work opens a new avenue for the non-invasive assessment of regional lung mechanics. The proposed approach complements existing imaging and functional tests by providing access to physically interpretable mechanical properties that are not directly observable through static images or global pulmonary indices.

An important implication of this framework is its natural compatibility with data-driven analysis. Because the measured frequency responses and recovered model parameters are grounded in a physical description of lung mechanics, they form structured, low-dimensional features that are well-suited for machine-learning–based classification. Rather than applying artificial intelligence directly to raw signals or images, learning algorithms can operate on physics-informed representations, enabling the discrimination of disease-specific mechanical patterns while preserving interpretability.

Future work will focus on experimental validation, refinement of the mechanical model, and exploration of clinical use cases. In parallel, the integration of physics-based system identification with machine-learning classifiers may enable automated, robust recognition of pathological mechanical signatures and longitudinal tracking of disease progression. More broadly, the proposed framework illustrates how classical principles of dynamics and inverse problem theory, combined with modern data-driven methods, can be leveraged to develop new diagnostic models in medicine.

Potential clinical use cases include longitudinal monitoring after thoracic or upper-abdominal surgery, regional mechanical assessment on mechanically ventilated ICU patients, and tracking of mechanical adaptation during pulmonary rehabilitation in chronic respiratory disease. These scenarios are attractive because they may benefit from repeated non-invasive measurements and from physically interpretable markers of regional stiffness and coupling.

### 4.10. Clinical Perspective

What is new? This study introduces a diagnostic model that treats the lung–thorax system as a mechanical dynamical system and infers regional lung mechanical properties from externally measured frequency responses. Unlike conventional imaging or global pulmonary function tests, this approach identifies pathology through changes in the system’s dynamical signature rather than through static structural features or scalar indices.

What are the clinical implications? If validated experimentally, this framework could provide a non-invasive means of assessing regional lung mechanics using low-amplitude external excitation applied at the chest wall. Such an approach may complement existing diagnostic tools by offering physically interpretable information about tissue stiffness and heterogeneity, potentially enabling the earlier detection of mechanical alterations and longitudinal monitoring without reliance on imaging. In the setting of pulmonary rehabilitation, physics-based estimates of regional stiffness and coupling could help identify patients with distinct mechanical phenotypes, support more individualized prescription of rehabilitation intensity and modality, and provide an objective mechanical marker to accompany conventional functional and symptomatic outcomes.

What is the next step? Future work will focus on experimental validation using physical lung phantoms and human studies, refinement of the mechanical model, and evaluation of feasibility in clinical settings including in patients undergoing pulmonary rehabilitation.

## 5. Conclusions

In this work, we proposed and evaluated a mechanical system-identification framework for lung assessment, in which internal mechanical properties are inferred from externally measured frequency responses. Using a multi-degree-of-freedom viscoelastic model, we demonstrated that localized stiffness alterations produce distinct and identifiable signatures in the global frequency response of the lung–thorax system. Sensitivity analysis and Fisher-information evaluation further showed that the governing stiffness parameters are locally identifiable within the selected frequency band and are not affected by numerical degeneracy in the examined parameter subspace.

The results show that regional mechanical parameters can be recovered through inverse analysis and that frequency response features enable reliable discrimination between different pathological configurations. Importantly, these findings establish the feasibility of a diagnostic model grounded in classical mechanics and inverse problem theory, rather than direct imaging or purely data-driven classification.

While the present study is based on simulations, it defines a physically interpretable framework for a non-invasive diagnostic paradigm that complements existing lung assessment methods. Because the diagnostic information is encoded in structured, physically meaningful features, the framework is naturally compatible with machine-learning approaches for automated interpretation without sacrificing interpretability. Robustness analyses incorporating controlled measurement noise and inter-subject mechanical variability confirmed that classification performance degrades gradually rather than catastrophically under increasing uncertainty, indicating that discriminative structure arises from stable dynamical signatures rather than fragile feature separability. With further experimental validation and model refinement, mechanical system identification may provide a new avenue for assessing regional lung mechanics in clinical practice, and may ultimately contribute to more personalized planning and monitoring of pulmonary rehabilitation in chronic respiratory disease.

The immediate priority is staged validation in controllable thoracic phantoms followed by pilot human feasibility measurements.

## Figures and Tables

**Table 1:** Mechanical degrees of freedom used in the thoracic system-identification model. The four-degree-of-freedom (4-DOF) representation assigns each dynamical variable to a major mechanical compartment of the thorax, including the chest wall and the principal lung regions, enabling simplified yet physiologically meaningful modeling of respiratory mechanics.

Degree of Freedom	Physical Compartment
DOF 0	Chest wall (sternum, ribs, and superficial tissues)
DOF 1	Right upper lung region
DOF 2	Right lower lung region
DOF 3	Left lung (lumped)

**Table 2:** Elastic coupling structure of the thoracic mechanical model. The table summarizes the spring elements used to construct the stiffness matrix of the four-degree-of-freedom (4-DOF) thoracic model. Each spring represents an elastic interaction between anatomical compartments, including chest wall–lung coupling, intra-pulmonary mechanical interactions, mediastinal coupling between lungs, and external thoracic support. These elastic connections define the entries of the global stiffness matrix and allow localized pathological alterations—such as increased stiffness in fibrosis or reduced stiffness in emphysema—to be incorporated as perturbations of specific spring constants.

Spring	Physical Interpretation
$k_{01}$	Chest wall ↔ right upper lung
$k_{02}$	Chest wall ↔ right lower lung
$k_{03}$	Chest wall ↔ left lung
$k_{12}$	Right upper ↔ right lower lung
$k_{13}$	Right upper ↔ left lung (mediastinal coupling)
$k_{23}$	Right lower ↔ left lung
$k_{0g}$	Chest wall ↔ external support (spine/body)

**Table 3:** Classification performance metrics.

Condition	Precision	Recall	F1-Score	Support
Healthy	1.00	1.00	1.00	9
Fibrosis RU	1.00	1.00	1.00	9
Fibrosis RL	1.00	1.00	1.00	9
Accuracy			1.00	27

## Data Availability

All relevant data are contained within the manuscript.

## Supplementary Materials

The following supporting information can be downloaded at: https://www.mdpi.com/article/10.3390/life16040555/s1, Figure S1: Robustness of classification to measurement noise; Figure S2: Effect of inter-subject stiffness variability on feature structure; Figure S3: Combined effect of measurement noise and stiffness variability.
