# Supplementary material for: A Simulation-Based Mechanical System-Identification Framework for Non-Invasive Lung Diagnostics and Personalized Pulmonary Rehabilitation
Source: Life (Basel). 2026 Mar 27;16(4):555. doi: 10.3390/life16040555 (PMC13118028; doi:10.3390/life16040555)
Supplement: Supplementary file 1 [file life-16-00555-s001.zip › life-4208960-supplementary.pdf]

Supplementary Results

S1. Robustness of classification to measurement noise

To evaluate the sensitivity of the proposed diagnostic framework to measurement noise, classification performance was assessed under increasing levels of multiplicative noise applied to the frequency-response magnitudes. Noise was modeled as independent zero-mean Gaussian perturbations applied to each frequency point, scaled relative to the response magnitude, consistent with moderate experimental variability.

Four noise levels were considered: σ = 0.00, 0.03, 0.10, and 0.20 (relative). For each noise level, frequency-response data were simulated for three conditions (healthy, fibrosis RU, fibrosis RL), with 50 realizations per class. Dimensionality reduction was performed using principal component analysis (PCA), retaining six components, followed by multinomial logistic regression classification. To obtain statistically robust estimates, the entire train–test–classify procedure was repeated 30 times using different random splits, and mean ± standard deviation classification accuracy was computed.

Supplementary **Figure S1** shows classification accuracy as a function of noise level. Performance remained near perfect for low to moderate noise ($\sigma \leq 0.10$), with mean accuracy exceeding 0.99. Even at the highest noise level tested (σ = 0.20), accuracy remained above 0.98, with only a modest increase in variability across trials.

These results demonstrate that the frequency-response features used by the model are highly robust to realistic levels of measurement noise, and that diagnostic performance degrades gracefully rather than catastrophically as noise increases.

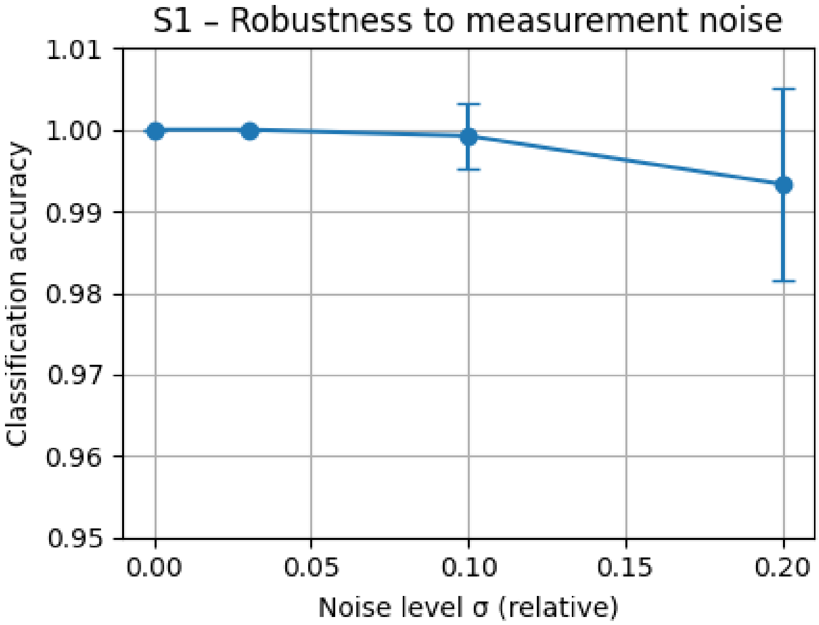

Supplementary Figure S1. Classification accuracy (mean ± standard deviation over 30 trials) as a function of measurement noise level σ. Accuracy remains close to unity for $\sigma \leq 0.10$ and remains well above 0.98 even at σ = 0.20.

S2. Effect of inter-subject stiffness variability on feature structure

To assess the impact of physiological inter-subject variability, stiffness parameters were allowed to vary randomly across realizations within each diagnostic class. For each non-ground spring element, stiffness values were perturbed according to

$$k_{ij}^{(var)} = k_{ij}(1+\epsilon), \quad \epsilon \sim \mathcal{N}(0, \sigma_k)$$

with relative stiffness variability $\sigma_k = 0.10$ (10%). Ground stiffness terms were kept fixed. Mean stiffness values corresponded to a stiffening factor of 1.8 for fibrosis RU and fibrosis RL relative to the healthy reference. Measurement noise with $\sigma = 0.03$ was included.

For each class, 50 realizations were generated. Frequency-response magnitude vectors (all DOFs, full frequency grid) were assembled and projected into a lower-dimensional space using PCA. For visualization, only the first two principal components were retained.

The explained variance ratios of the first two principal components were:

- PC1: 46.99%
- PC2: 24.43%

Together, PC1 and PC2 account for approximately 71% of the total variance in the frequency-response features.

Supplementary **Figure S2** shows the projection of all realizations onto the PC1–PC2 plane. Despite the presence of 10% stiffness variability and measurement noise, the three diagnostic classes form clearly separated clusters. Healthy realizations cluster near the origin, while fibrosis RU and fibrosis RL occupy distinct regions primarily separated along PC1.

Within each class, stiffness variability manifests as elongation and spread of the clusters, but does not produce substantial overlap between pathological configurations. This indicates that the dominant variance captured by PCA reflects disease-related mechanical changes rather than random inter-subject variability.

These results show that the discriminative structure of the frequency-response features is preserved under physiologically plausible parameter variability, supporting the robustness of the proposed diagnostic framework.

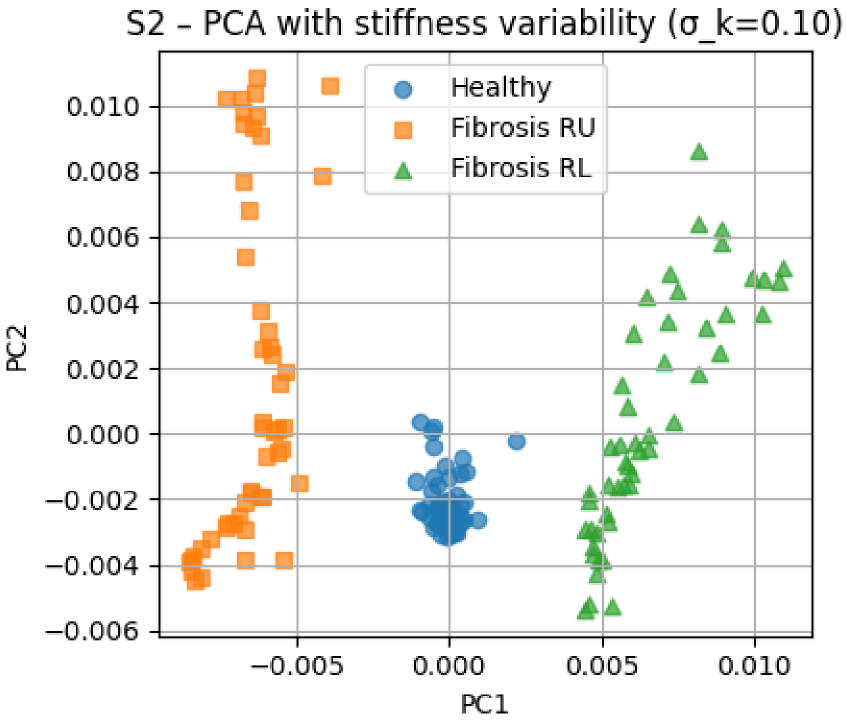

Supplementary Figure S2. PCA projection of frequency-response features under inter-subject stiffness variability ($\sigma_k = 0.10$) and measurement noise ($\sigma = 0.03$). Each point corresponds to a single realization. PC1 and PC2 explain 46.99% and 24.43% of the total variance, respectively. Clear separation between healthy, fibrosis RU, and fibrosis RL configurations is preserved despite variability.

S3. Combined effect of measurement noise and stiffness variability

Finally, robustness was evaluated under the combined presence of measurement noise and inter-subject stiffness variability. Stiffness variability was fixed at $\sigma_k = 0.10$, while measurement noise was varied across $\sigma = 0.03$, 0.10, and 0.20. For each noise level, 50 realizations per class were simulated, and classification was repeated 30 times using different random train–test splits.

Supplementary **Figure S3** shows the mean ± standard deviation classification accuracy as a function of noise level. A horizontal dashed line indicates chance-level performance (1/3) for the three-class problem.

Classification accuracy remained well above chance across all noise levels tested. At $\sigma = 0.03$ and $\sigma = 0.10$, mean accuracy exceeded 0.80, indicating reliable discrimination even under combined uncertainty. At $\sigma = 0.20$, accuracy decreased modestly but remained substantially above chance, with no collapse in performance.

These results demonstrate that the diagnostic signatures identified by the mechanical system-identification framework are resilient to simultaneous measurement noise and physiological variability. Importantly, performance degrades smoothly rather

than abruptly, suggesting that the model operates in a stable regime suitable for real-world conditions.

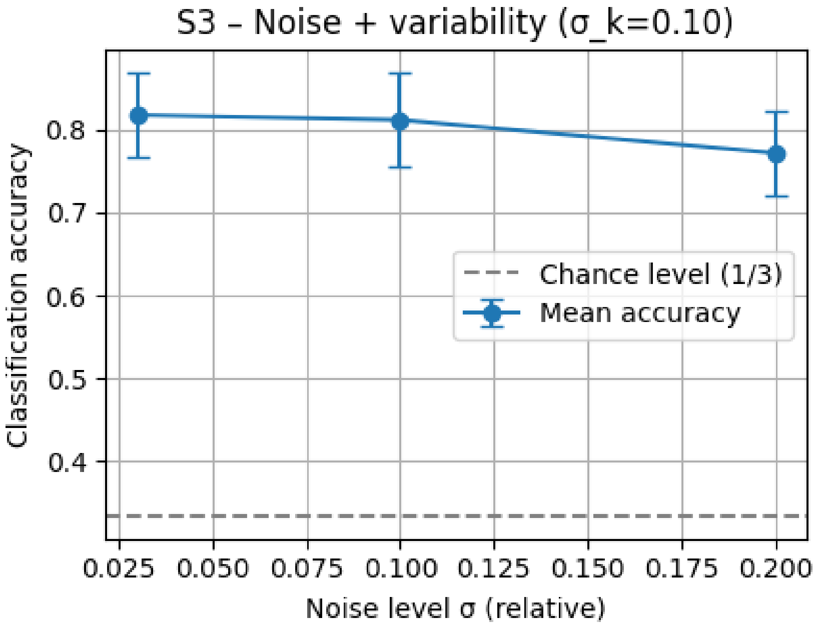

Supplementary Figure S3. Classification accuracy (mean ± standard deviation over 30 trials) under combined stiffness variability (σ_k = 0.10) and increasing measurement noise. The dashed line indicates chance-level performance (1/3). Accuracy remains well above chance across all noise levels tested.

Supplementary summary

Together, the supplementary experiments demonstrate that:

1. Classification based on frequency-response features is highly robust to measurement noise.
2. Physiologically plausible inter-subject stiffness variability broadens feature distributions but preserves class separability.
3. Even under combined noise and variability, diagnostic performance remains well above chance.

These findings support the feasibility of extending the proposed mechanical system-identification framework from idealized simulations toward realistic experimental and clinical scenarios.
